# Supplementary material for: Content-rich biological network constructed by mining PubMed abstracts
Source: BMC Bioinformatics. 2004 Oct 8;5:147. doi: 10.1186/1471-2105-5-147 (PMC528731; doi:10.1186/1471-2105-5-147)
Supplement: Additional File 2 — The original results of the above study (non-essential files are deleted to keep the file size under the limit set by BMC bioinformatics). [file 1471-2105-5-147-S2.bz2 › chilibotAdditionalFile2/dip20/122ID8668348E466/html/MAP2K1_BRAF.html]

 


 **MAP2K1** and **BRAF** 
  
Found 75 abstracts in PubMed, retrieved 20.  
 

 What does Google say? 
 PDF only 
| .edu only 

---

**Interactive relationship** (e.g. stimulation, inhibition, etc)

**Inhibitory relationship**- We found that 8 bromoadenosine cyclic monophosphate 8Br cAMP induced the activation of ERK and mitogen activated protein kinase MEK  [ **MAP2K1** ] , but inhibited B Raf  [ **BRAF** ]  kinase via a protein kinase A PKA mediated mechanism.  Ref: 12479852 Leuk Res, 2003
**Neutral relationship**- In contrast, B Raf  [ **BRAF** ] , a MAPKKK specific for the MEK  [ **MAP2K1** ]  ERK module, binds PA28gamma and a.  Ref: 12650640 Biochem J< MedlineTA>Biochem J, 2003
- Raf comprises a family of three kinases, A Raf, B Raf  [ **BRAF** ]  and Raf 1, which are best known as key regulators of the MEK  [ **MAP2K1** ]  MAPK ERK cascade.  Ref: 11950876 J Cell Sci, 2002

**Non-interactive relationship** (e.g. studied together, co-existance, homology, etc.)

- GLP1 stimulated activation of Erk is blocked by inhibitors of MEK  [ **MAP2K1** ] , but GLP1 does not induce the activation of A Raf, B Raf  [ **BRAF** ] , C Raf, or Ras.  Ref: 12364324 J Biol Chem, 2002
- Surprisingly, in contrast to proliferative cells, we found that the serine threonine kinases Raf 1 and B Raf  [ **BRAF** ]  were not an intermediate kinase between conventional PKCs and MEK1  [ **MAP2K1** ]  2.  Ref: 12435596 FEBS Lett, 2002
- Disruption of the Raf MEK  [ **MAP2K1** ]  ERK MAPK kinase pathway, either by RAS or  **BRAF**  mutation, was detected in approximately 62% of all CC and is therefore one of the most frequent defects in cholangiocellular carcinogenesis.  Ref: 12692057 Gut, 2003
- Down stream of PKA, the activity of B Raf  [ **BRAF** ]  was significantly decreased by nicotine in SH SY5Y cells, as determined by direct measurement of MEK1  [ **MAP2K1** ]  phosphorylation or in vitro kinase assays.  Ref: 11905997 J Neurochem, 2002
- After immunoprecipitation of Raf 1 and B Raf  [ **BRAF** ] , the basal glutathione S transferase MEK1  [ **MAP2K1** ]  phosphorylation observed in resting platelets was not upregulated by thrombin and was still observed in the absence of anti Raf 1 or anti B Raf  [ **BRAF** ]  antibodies.  Ref: 12435596 FEBS Lett, 2002
- To investigate the importance of Raf MEK  [ **MAP2K1** ]  ERK signaling in human hematopoietic cell growth, the effects of three different Raf genes, A Raf, B Raf  [ **BRAF** ]  and Raf 1, on cell cycle progression and regulatory gene expression were examined in TF 1 cells transformed to grow in response to beta estradiol regulated DeltaRaf ER genes.  Ref: 12429936 Cell Cycle< MedlineTA>Cell Cycle, 2002
- Taken together, our results identify a novel mechanism whereby a Gi o protein coupled receptor inhibits cell proliferation by stimulating ERK signaling via a SHP 1 SHP 2 PI3K Ras Rap1 B Raf  [ **BRAF** ]  MEK  [ **MAP2K1** ]  pathway.  Ref: 12878607 J Biol ChemJ Biol Chem, 2002
